# Recognition of Voltage Sag Sources Based on Phase Space Reconstruction and Improved VGG Transfer Learning

**DOI:** 10.3390/e21100999

**Published:** 2019-10-12

**Authors:** Yuting Pu, Honggeng Yang, Xiaoyang Ma, Xiangxun Sun

**Affiliations:** 1 College of Electrical Engineering, Sichuan University, Chengdu 610065, China; 2017223030026@stu.scu.edu.cn (Y.P.); pqlabqq@126.com (H.Y.); 2 China Mobile (Hangzhou) Information Technology Co., Ltd., Hangzhou 311100, China; sunxiangxun@cmhi.chinamobile.com

**Keywords:** phase space reconstruction, VGG, transfer learning, voltage sag source, attention, cross entropy

## Abstract

The recognition of the voltage sag sources is the basis for formulating a voltage sag governance plan and clarifying the responsibility for the accident. Aiming at the recognition problem of voltage sag sources, a recognition method of voltage sag sources based on phase space reconstruction and improved Visual Geometry Group (VGG) transfer learning is proposed from the perspective of image classification. Firstly, phase space reconstruction technology is used to transform voltage sag signals, generate reconstruction images of voltage sag, and analyze the intuitive characteristics of different sag sources from reconstruction images. Secondly, combined with the attention mechanism, the standard VGG 16 model is improved to extract the features completely and prevent over-fitting. Finally, VGG transfer learning model uses the idea of transfer learning for training, which improves the efficiency of model training and the recognition accuracy of sag sources. The purpose of the training model is to minimize the cross entropy loss function. The simulation analysis verifies the effectiveness and superiority of the proposed method.

## 1. Introduction

In recent years, with the widespread use of power electronic devices in power grid and sensitive devices in industrial production, the impact of voltage sag has gradually attracted attention in the electrical field. Accurate recognition of the source of voltage sag can help the timely formulation of the governance plan and the clear division of the responsibilities of both parties in the accident, effectively reducing economic losses and resolving related disputes [1].

At present, the research on the recognition of voltage sag sources falls into two categories: direct methods [2,3,4,5,6,7] and indirect methods [8,9,10,11,12,13,14,15,16,17,18,19]. The direct methods include the RMS method [2,3] and the deep learning method [4,5,6,7]. Indirect methods include two parts: feature extraction and pattern recognition. Common methods for feature extraction include wavelet transform [8,9], Fourier transform [10,11], S transform [12], Hilbert transform [13], and empirical mode decomposition [14], etc. The main methods of pattern recognition include neural network [15,16], support vector machine [17], principal component analysis [18], fuzzy comprehensive evaluation [19], and so on. Among them, the RMS method [2,3] is simple and easy to implement, but it is easy to produce misjudgment for complex sag situations; the deep learning method [4,5,6,7] does not rely on manual extraction of features, but the model training efficiency is low. In the feature extraction process of indirect methods, the mathematical models are mature, the features are clear. However, indirect methods are limited to two-dimensional plane characteristics, and there are still some shortcomings: wavelet transform [8] is based on weak signal interference and good frequency domain characteristics, otherwise the correct rate will be seriously affected; the time-frequency localization property of short-time Fourier transform [10] is poor, so it cannot well reflect the transient characteristics of voltage change. S-transform [12] has low resolution when analyzing signals with harmonic components. In the pattern recognition process of indirect methods, the neural network method [15] needs to manually set the characteristics which need to be extracted in advance and then use the classifier to classify, relying too much on expert experience to discriminate rules; the recognition results of multi-classifier support vector machine are susceptible to thresholds [17]; the principal component analysis method [18] selects the main feature quantity, and although the number of feature quantities is reduced, the information contained in part of the sample data is lost.

In order to overcome the above problems, from the perspective of image classification, firstly, this paper proposes to use phase space reconstruction technology to extract the characteristics of voltage sag and map voltage sag signals into high-dimensional space [20]. The high-dimensional space image of sag keeps the complete information. By highlighting the characteristics reflected in high-dimensional space, it is obvious and easy to recognize different voltage sag sources. Then, the Visual Geometry Group (VGG) 16 model [21] is improved. There are a lot of research achievements about VGG model in the field of image classification, and its classification effect is good, so it is widely used [22,23,24]. Before the VGG model automatically extracts features, the attention mechanism is added to make the feature description more complete, which further prevents over-fitting and improves the generalization ability of the model [25]. Attention mechanism in deep learning simulates the attention model of human brain. At present, it is very popular and widely used in machine translation [26], speech recognition [27], image caption [28], and many other fields. In addition, VGG transfer learning model is constructed by using the idea of transfer learning [29], which can improve the efficiency of network training and the recognition accuracy of voltage sag sources.

The rest of this paper is organized as follows. In Section 2, feature extraction of different kinds of voltage sags is analyzed with phase space reconstruction theory. The theory of VGG modeling with attention mechanism and voltage sag recognition method with transfer learning are presented in Section 3. In Section 4, some simulation data are used to verify the effectiveness of the proposed method. Conclusions are drawn in Section 5.

## 2. Voltage Sag Source Feature Extraction Based on Phase Space Reconstruction Theory

### 2.1. Classification of Voltage Sag Sources

At present, there are three main causes of voltage sag: short circuit fault, large induction motor starting, and unloaded transformer energizing.

- Short circuit fault is the main cause of voltage sag. Different short circuit faults can cause different sags. The voltage sag caused by three phase short circuit fault is equal in three-phase voltage magnitude. The three-phase magnitude of voltage sag caused by other short circuit types is different. Voltage swell may occur while sag occurs in an asymmetric short circuit. At the beginning and end of the voltage sag, the magnitude suddenly changes, and there is no change in the voltage magnitude during the sag.
- When a large induction motor is starting, it will draw much larger current from the power supply than normal operation. The typical starting current is 5-6 times the rated working current, thus resulting in voltage sag. When the sag occurs, the three-phase voltage drops at the same time, and the sag magnitude is basically the same. There is no sudden change in the recovery process, and it is gradually recovered.
- Because of the saturation characteristic of the core, the inrush current of transformer when switched on and off is several times the rated current, which will cause voltage sag. The initial phase angle of three-phase voltage always differs by 120 degrees, so the magnitude of three-phase sag is always unbalanced. Large transformers usually need dozens of cycles to recover because of their small resistance and large reactance. In addition, the voltage waveform of sag contains higher harmonics.

For short circuit faults, according to the fault phases, they are divided into seven types: A_1a_, A_1b_, A_1c_, A_2ab_, A_2ac_, A_2bc_, and A_3abc_ which respectively represent A-phase short circuit, B-phase short circuit, C-phase short circuit, A- and B-phase short circuit, A- and C-phase short circuit, B- and C-phase short circuit, and three phase short circuit fault. For the starting of induction motor, it is recorded as B_1_. For large transformer energizing, it is recorded as C_1_. So, there are nine types of voltage sag sources.

### 2.2. Phase Space Reconstruction Theory

Phase space reconstruction theory holds that the development process of any component in a dynamic system implies information of other relevant components, and the original change rule of the system can be extracted and restored by analyzing the time series data of a component.

For one-dimensional time series *x* = {*x*_1_, *x*_2_, ..., *x_N_*}, according to Takens’ delay time embedding theory [30], one-dimensional time series is extended to high-dimensional space by using two reconstruction parameters (delay time *τ* and embedding dimension *m*):

(1) $$X=\left[\begin{array}{c} X_{1}\\ X_{2}\\ \vdots \\ X_{M} \end{array}\right]=\left[\begin{array}{cccc} x_{1} & x_{1+\tau } & \cdots & x_{1+\left(m-1\right)\tau }\\ x_{2} & x_{2+\tau } & \cdots & x_{2+\left(m-1\right)\tau }\\ \vdots & \vdots & \ddots & \vdots \\ x_{M} & x_{M+\tau } & \cdots & x_{M+\left(m-1\right)\tau } \end{array}\right]=\left[\begin{array}{cccc} U_{1} & U_{2} & \cdots & U_{m} \end{array}\right]$$

where column vectors *U_k_* (*k* = 1, 2, ..., *m*) represent coordinates of each dimension, line vectors *x_k_* (*k* = 1, 2, ..., *M*) constitute the phase points in the phase space, ***X*** is a matrix of *M* × *m*, and *X_k_* is a vector of 1 × *m*, *τ* represents delay time which is the sampling interval of time series, *M* = *N* − (*m* − 1)*τ*. These *M* points together constitute the phase trajectory of reconstruction phase space from voltage sag time series.

In order to ensure the visibility of the reconstruction images, *m* can take 1, 2, 3. At the same time, the larger *m* is, the larger the dimension of phase space is, the more information it contains. Therefore, *m* = 3 is the most appropriate. When *m* = 3, Equation (1) becomes

(2) $$X=\left[\begin{array}{c} X_{1}\\ X_{2}\\ \vdots \\ X_{M} \end{array}\right]=\left[\begin{array}{ccc} x_{1} & x_{1+\tau } & x_{1+2\tau }\\ x_{2} & x_{2+\tau } & x_{2+2\tau }\\ \vdots & \vdots & \vdots \\ x_{M} & x_{M+\tau } & x_{M+2\tau } \end{array}\right]=\left[\begin{array}{ccc} U_{1} & U_{2} & U_{3} \end{array}\right]$$

With C-C method [31], we determine *τ* = 2. So, Equation (2) becomes

(3) $$X=\left[\begin{array}{c} X_{1}\\ X_{2}\\ \vdots \\ X_{M} \end{array}\right]=\left[\begin{array}{ccc} x_{1} & x_{3} & x_{5}\\ x_{2} & x_{4} & x_{6}\\ \vdots & \vdots & \vdots \\ x_{M} & x_{M+2} & x_{M+4} \end{array}\right]=\left[\begin{array}{ccc} U_{1} & U_{2} & U_{3} \end{array}\right]$$

In this paper, the sampling interval is 1/1000 s, so *τ* = 2 means 2/1000 s.

### 2.3. Phase Space Reconstruction of Different Voltage Sag Signals

**Figure 1:**
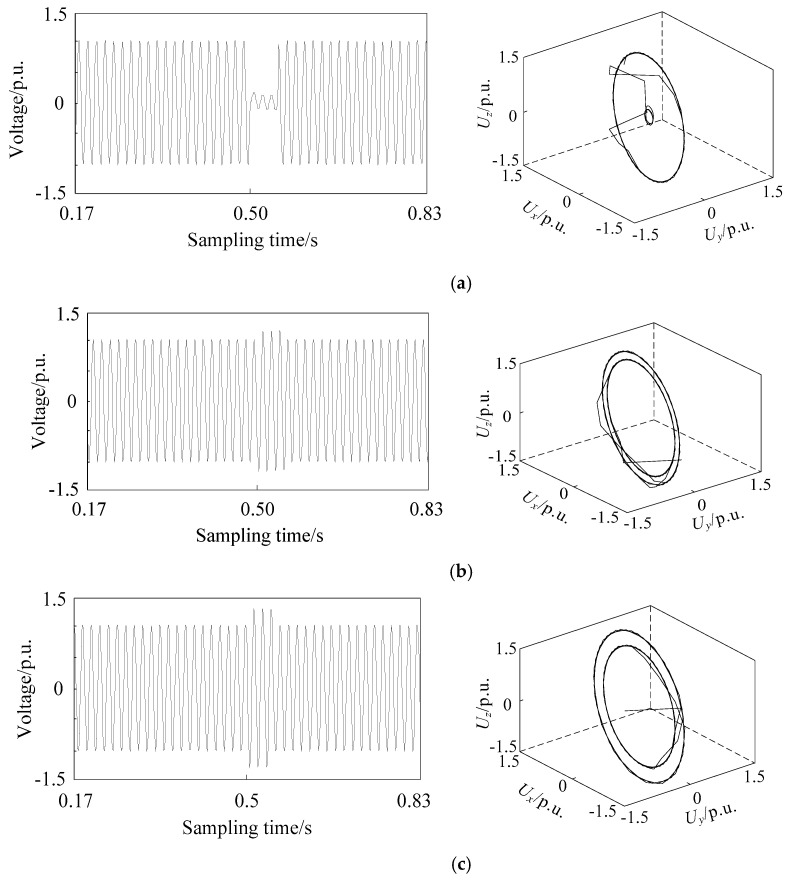
Voltage sag signal waveform (**left**) and corresponding phase space reconstruction image (**right**) of A-phase short circuit fault in phase (**a**) A, (**b**) B, and (**c**) C.

**Figure 2:**
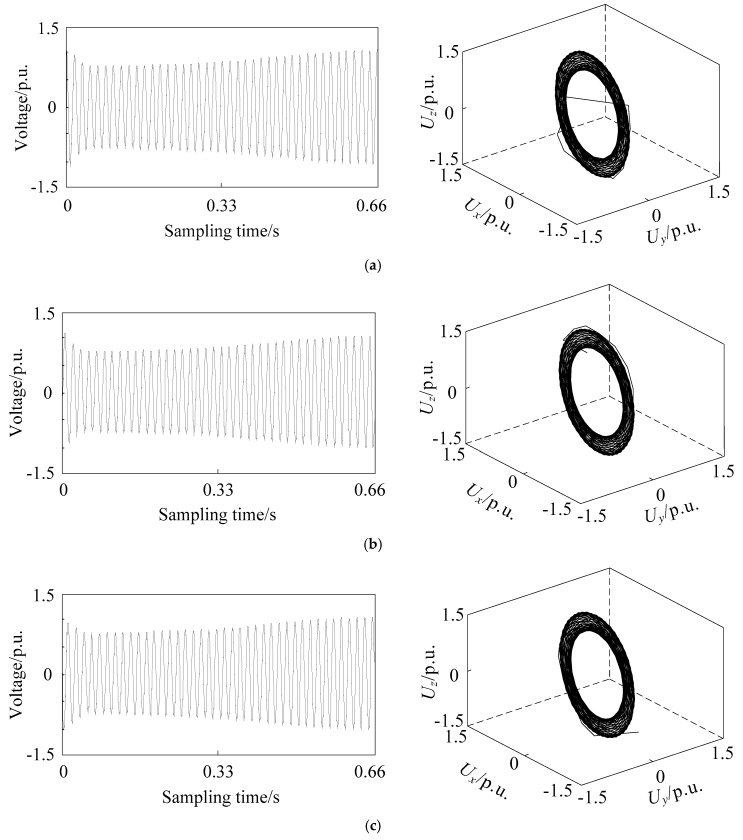
Voltage sag signal waveform (**left**) and corresponding phase space reconstruction image (**right**) of large induction motor starting in phase (**a**) A, (**b**) B, and (**c**) C.

**Figure 3:**
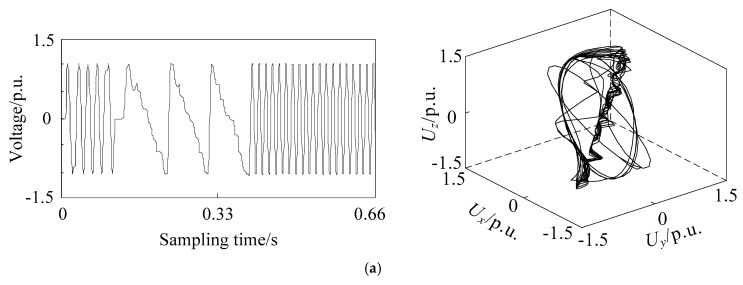

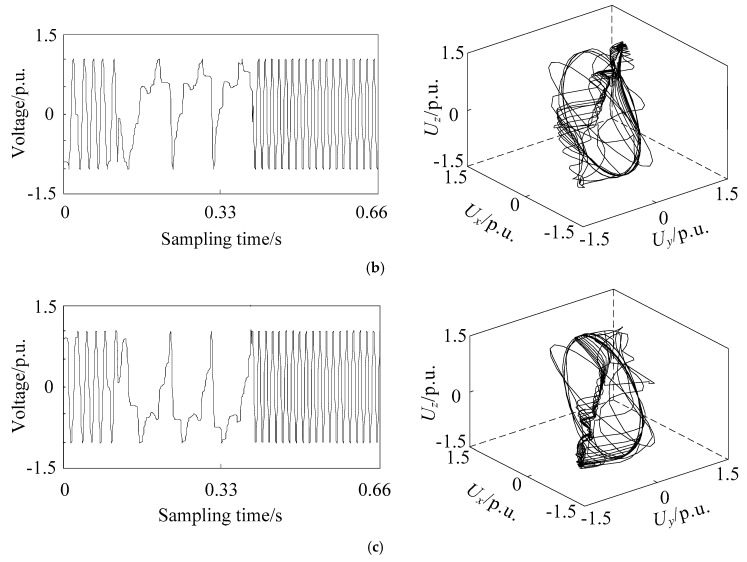
Voltage sag signal waveform (**left**) and corresponding phase space reconstruction image (**right**) of unloaded transformer energizing in phase (**a**) A, (**b**) B, and (**c**) C.

The basic frequency of the simulation model is set to 50 Hz, and the total simulation time is set to 1 s. The sampling frequency is set to 1 kHz, so the sampling point of voltage signal is 1000. The phase space reconstruction of voltage sag signal caused by single phase short circuit fault, large induction motor starting and unloaded transformer energizing is carried out. The three-dimensional coordinates are recorded as *U_x_*, *U_y_*, and *U_z_*, respectively, which equal to *U_1_*, *U_2_*, and *U_3_*. The reconstructed images are shown in Figure 1, Figure 2 and Figure 3. The x-coordinate of sampling time is adjusted by omitting 0.34 s when no sag occurs to obtain better quality, while the sampling time of reconstructed image is still one second.

From Figure 1, Figure 2 and Figure 3, it can be seen that the attractor of stable sinusoidal waveform is in the form of a limit cycle in phase space, and the size of the limit cycle represents the size of sinusoidal wave. For example, when the single phase short circuit fault occurs in Figure 1, the magnitude of phase A decreases, while there is a very small limit cycle in the corresponding reconstruction image; when the magnitude of phase B and C increases, there is a corresponding larger limit cycle in the reconstruction image. A larger limit cycle can be used to judge the type of fault as A-phase short circuit fault. The voltage changes slowly when the induction motor is starting. So, in Figure 2, many limit cycles are generated, and there is no sudden change between the cycles. The most special one is Figure 3: in addition to limit cycles, there are strange attractors in the reconstruction image of transformer energizing, which are caused by high-order harmonics in voltage signals. Therefore, the identification of voltage sag sources based on phase space reconstruction image has the following main characteristics:

- The number of limit cycles
- The size of limit cycles
- The existence of strange attractors
- The number of mutation trajectories

In the case of short circuit fault, the duration of voltage sag is shown as overlap of phase trajectory on phase space reconstruction image, which does not affect its identification. The phase space reconstruction of voltage sag retains the complete sag information and is more intuitive and significant than the original signal waveform.

## 3. Recognition of Voltage Sag Source Based on VGG

### 3.1. VGG Network Structure

VGG is a deep convolution neural network developed by researchers from the Visual Geometry Group of Oxford University and Google DeepMind Company. VGG 16 is a classical algorithm for image classification [21].

**Figure 4:**
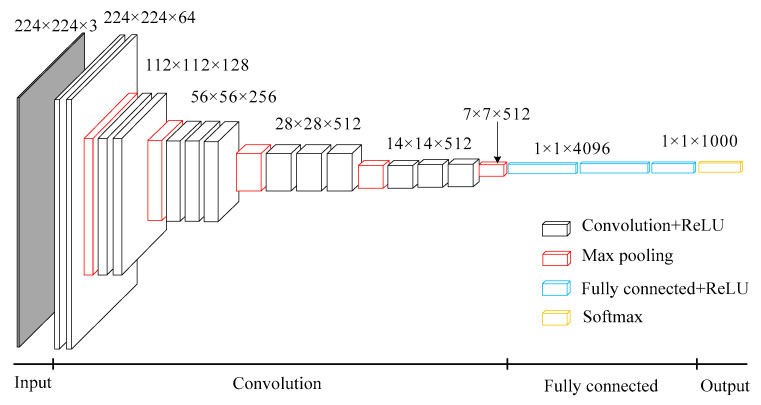
Standard VGG 16 network structure. 224 × 224 × 3 and so on represent the dimensions of matrix. Convolution + ReLU means ReLU function activation after convolution operation. Fully connected + ReLU means ReLU function activation after fully connected operation.

As shown in Figure 4, VGG 16 is simple in structure, consisting of 13 convolution layers, 5 pooling layers, 3 fully connected layers, and 1 softmax output layer. All activation units of hidden layers adopt Rectified Linear Units (ReLU) function. The convolution kernels are 3 × 3 and the pooling kernels are 2 × 2. Among them, VGG 16 uses several convolution layers with smaller convolution kernels (3 × 3), which on one hand can reduce parameters, and on the other hand, it is equivalent to more non-linear mappings, which can increase the fitting ability of the network. The number of channels in the first layer of VGG 16 network is 64, and the number of channels in each layer of VGG16 network is doubled, up to 512 channels. With the increase of the number of channels, more information can be extracted. In addition, the convolution kernel focuses on enlarging the number of channels and the pooling kernel focuses on narrowing the width and height, which make the model deeper and wider in structure, and control the amount of calculation at the same time.

The supervised pre-training of VGG can be divided into two processes: forward propagation and backward propagation. Forward propagation calculates input characteristics at each level as follows:

(4) $$x^{\left(l\right)}=f\left(w^{\left(l\right)}x^{\left(l-1\right)}+b^{\left(l\right)}\right)$$

where *l* is the current layer, ***x***^(*l*–1)^ is the input of the layer, ***x***^(*l*)^ is the output of the layer, ***w*** is the weight, ***b*** is the bias, *f*(·) is the ReLU function. The equation of the ReLU function is

(5) f(x)=max(0,x)

Convolution operation is a linear operation. In order to increase the non-linear ability of the neural network and consider the training complexity of the network, ReLU is used as an activation function to perform nonlinear transformation on the feature after linear operation.

When backward propagation occurs, the parameters *w_ij_*^(*l*)^ and *b_i_*^(*l*)^ of each layer are updated by batch gradient descent method. The updated formulas are as follows:

(6) $$\left\{ \begin{array}{l} w_{ij}^{\left(l\right)}=w_{ij}^{\left(l\right)}-\alpha \frac{\partial }{\partial w_{ij}^{\left(l\right)}}J\left(w,b\right)\\ b_{i}^{\left(l\right)}=b_{i}^{\left(l\right)}-\alpha \frac{\partial }{\partial b_{i}^{\left(l\right)}}J\left(w,b\right) \end{array}\right.$$

where *α* is the learning rate, *i* is the *i*-th sample, *j* is the *j*-th mapping feature, and *J*(·) is the cross entropy loss function which will be mentioned in Section 3.2.

### 3.2. Cross Entropy Loss Function

Cross entropy is a concept in information theory [32]. It was originally used to estimate the average coding length. Given two probability distributions *p* and *q*, the cross entropy of *p* expressed by *q* is

(7) $$H\left(p,q\right)=-\sum_{x}p\left(x\right)\log q\left(x\right)$$

where *p*(*x*) is often used to describe the true distribution, *q*(*x*) is used to describe the distribution of model prediction in machine learning. The smaller the cross entropy *H*(*p*,*q*), the closer the two probability distributions are.

Information Entropy is the expectation of all information quantities:

(8) $$H\left(X\right)=-\sum_{x}p\left(x\right)\log p\left(x\right)$$

Relative entropy is also called Kullback–Leibler divergence. If there are two separate probability distributions *p*(*x*) and *q*(*x*) for the same random variable *x*, relative entropy can be used to measure the difference between the two distributions.

(9) $$\begin{array}{c} D_{KL}\left(p||q\right)=\sum_{x}p\left(x\right)\log\frac{p\left(x\right)}{q\left(x\right)}=\sum_{x}p\left(x\right)\log p\left(x\right)-\sum_{x}p\left(x\right)\log q\left(x\right)\\ =-H\left(X\right)+\left[-\sum_{x}p\left(x\right)\log q\left(x\right)\right]=H\left(p,q\right)-H\left(X\right) \end{array}$$

where the smaller the relative entropy *D_KL_*(*p*||*q*) is, the closer the two probability distributions are.

Relative entropy should be used to calculate the difference of probability distribution in calculating loss. However, the Equation (9) shows that:

(10) Relative entropy=Cross entropy−Information entropy

Since information entropy describes the amount of information needed to eliminate the uncertainty of *p* (the true distribution), its value should be minimum and fixed. Then, optimizing and reducing relative entropy is optimizing cross entropy, so it is easy and good to use cross entropy in machine learning.

Cross entropy loss function is as follows and the purpose of training is to minimize it:

(11) $$J\left(w,b\right)=-\frac{1}{m}\sum_{j=1}^{m}\sum_{i=1}^{n}y_{ji}\log h_{w,b}\left(x_{ji}\right)$$

where *n* is the number of categories, *m* is the number of samples, *y_ji_* is the true value of sample *j* in class *i*, and *h_w,b_*(*x_ji_*) is the predicted value of sample *j* in class *i*.

### 3.3. Attention Mechanism

Attention mechanism in deep learning simulates the attention model of human brain. At present, it is very popular and widely used in machine translation [26], speech recognition [27], image caption [28], and many other fields. Attention mechanism can select more key information from a large amount of information and suppress irrelevant information, so as to avoid the problem of over-fitting. Channel attention pays more attention to the global characteristics, while spatial attention has more prominent ability to control the local features, so the mixed attention called Convolutional Block Attention Module (CBAM), which combines channel and spatial dimension, can better depict the complete information [25]. 

**Figure 5:**
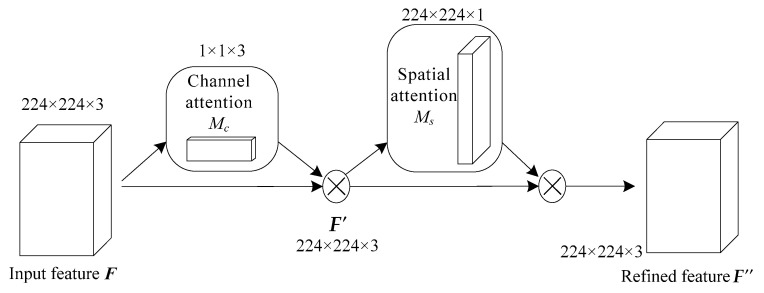
The whole attention module. 224 × 224 × 3 and so on represent the dimensions of matrix.

**Figure 6:**
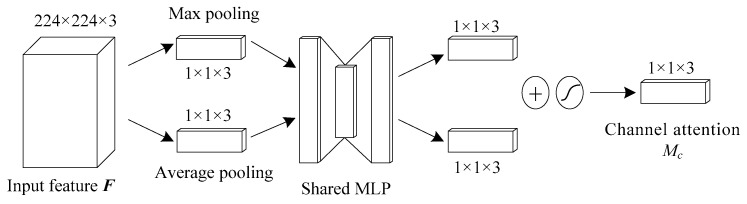
Channel attention module. 224 × 224 × 3 and so on represent the dimensions of matrix.

**Figure 7:**
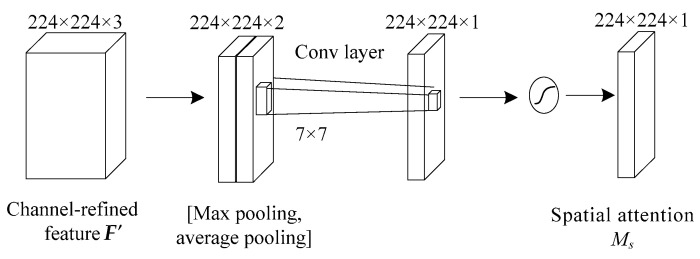
Spatial attention module. 224 × 224 × 3 and so on represent the dimensions of matrix.

When ***F*** is input, the attention module performs the following operations shown in Figure 5, Figure 6 and Figure 7:

(12) $$M_{c}\left(F\right)=\sigma \left(W_{1}\left(W_{0}\left(F_{\mathrm{avg}}\right)\right)+W_{1}\left(W_{0}\left(F_{\max}\right)\right)\right)$$

(13) $$F^{\prime }=M_{c}\left(F\right)⊗F$$

(14) $$M_{s}\left(F^{\prime }\right)=\sigma (f^{7\times 7}([F_{\mathrm{avg}}^{\prime };F_{\max}^{\prime }]))$$

(15) $$F^{\prime \prime }=M_{s}\left(F^{\prime }\right)⊗F^{\prime }$$

where *M_c_*(·) denotes attention extraction on channel dimension as shown in Figure 6, and *M_s_*(·) denotes attention extraction on spatial dimension as shown in Figure 7. ⊗ represents the multiplication of the corresponding position elements of two matrices, ***W***_0_ and ***W***_1_ represent the connection weight in the multi-layer perceptron (MLP) model using ReLU activation function, *σ* represents sigmoid activation function, ***F***_avg_ represents the vector ***F*** after average pooling operation and ***F***_max_ represents the vector ***F*** after maximum pooling operation, ***F’***_avg_ represents the vector ***F’*** after average pooling operation and ***F’***_max_ represents the vector ***F’*** after maximum pooling operation.

In the channel attention module, maximum pooling and average pooling are used simultaneously, and the parameters are shared by the shared MLP. The result of *M_c_*(·) operation can be obtained by summing up the results of fully connected layers after MLP. The input *F* is multiplied by the result of *M_c_*(·) operation to get the result *F*’ of channel attention, which is 224 × 224 × 3. 

In the spatial attention module, maximum pooling and average pooling are used for *F*’ simultaneously, and a vector with 2 channels is obtained by concatenating the two vectors of maximum pooling and average pooling. In order to get the importance of different pixels of feature maps on a plane, one convolution core is used to compress 2 channels into 1 channel. So, the convolution operation is carried out by using one 7 × 7 convolution kernel to ensure that the dimension of result of *M_s_*(·) operation is consistent with one channel, which is 224 × 224 × 1. 

In the end, the result of *M_s_*(·) operation is multiplied by the result of channel attention *F*’ to get the final vector of *F*’’. *F*’’ is consistent with *F* in dimension, which is 224 × 224 × 3.

**Figure 8:**
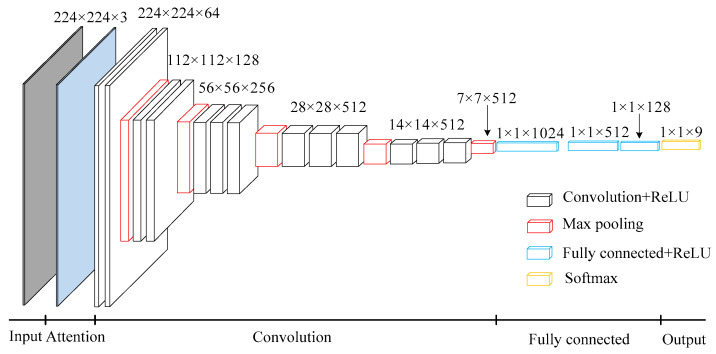
VGG16 network structure based on attention mechanism. 224 × 224 × 3 and so on represent the dimensions of matrix. Convolution + ReLU means ReLU function activation after convolution operation. Fully connected + ReLU means ReLU function activation after fully connected operation.

Adding the attention mechanism to the convolution layer, a VGG 16 model based on attention mechanism can be obtained, as shown in Figure 8. Because this paper divides the sources of sag into nine categories, the fully connected layers are fine-tuned to 1 × 1 × 128 and the Softmax layer changes into 1 × 1 × 9.

### 3.4. Voltage Sag Source Recognition Based on Improved VGG Transfer Learning

#### 3.4.1. Improved VGG Transfer Learning

Knowledge transfer is a hypothesis that can break down the same distributed samples and greatly increase the cross-domain ability of machine learning. The purpose of transfer learning [29] is to properly introduce existing knowledge into new fields, so that machines can acquire the ability to ‘draw inferences from one instance to another’. Based on the idea of transfer learning, the standard VGG 16 model is used to transfer the target task to the identification of voltage sag source, which greatly improves the efficiency and accuracy of training, and reduces the dependence on sample size. 

**Figure 9:**
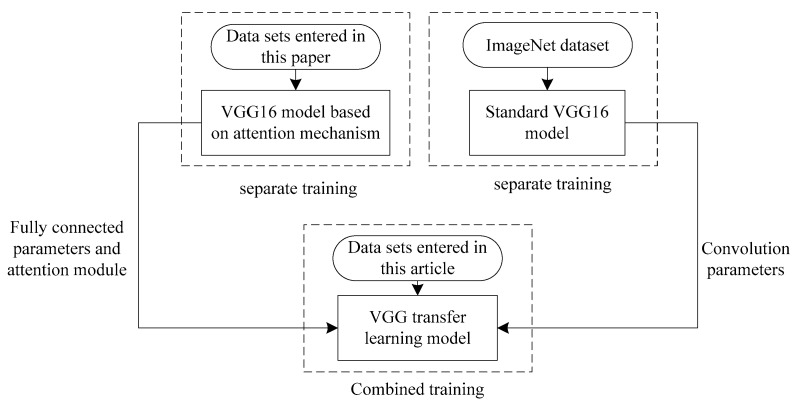
Training process of improved VGG transfer learning model.

ImageNet is a data set of more than 15 million images, about 22,000 categories [33]. A standard VGG 16 network has been pre-trained on the entire ImageNet dataset, using pre-computed weights. The complete VGG transfer learning model training process sketch is shown in Figure 9. Among them, the improved VGG transfer learning model is a combined model, which consists of standard VGG 16 and attention-based VGG 16 model. Firstly, the standard VGG 16 has been trained separately on the ImageNet dataset and fixed convolution parameters. Then, using VGG 16 based on attention mechanism, the input data set is trained separately, and the attention module and fully connected parameters are obtained. Finally, the standard VGG 16 and the attention-based VGG 16 model are combined for combined training. In this case, the combined training is for fine-tuning the results of separate training. 

In the training process of VGG 16 model based on attention mechanism, the voltage sag signal is transformed into reconstruction image according to the phase space reconstruction technology proposed in Section 2. Voltage sag sources are divided into nine categories, namely A_1a_, A_1b_, A_1c_, A_2ab_, A_2ac_, A_2bc_ A_3abc_, B_1_, and C_1_, which are used as labels of reconstruction images. Adam algorithm [34] is used to optimize the model parameters and adjust the learning rate adaptively.

#### 3.4.2. Voltage Sag Source Recognition Framework

**Figure 10:**
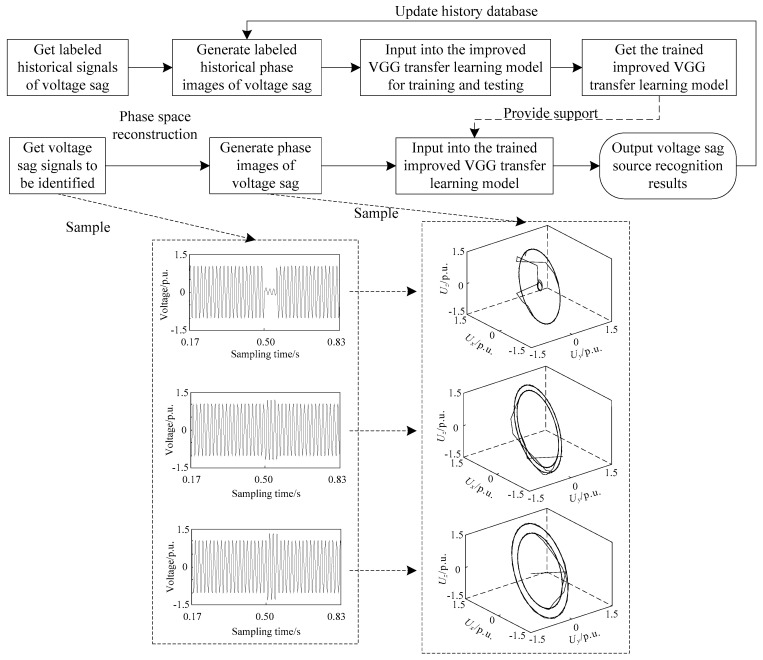
Voltage sag source recognition framework based on improved VGG transfer learning. The sample means an example of Figure 1.

The proposed framework for voltage sag source recognition based on improved VGG transfer learning is shown in Figure 10. The process is described as follows:

- Step 1: The historical data of voltage sag are read from the database. With the technology of phase space reconstruction referred in Section 2, historical reconstruction images of labeled different voltage sag sources can be generated.
- Step 2: As training and testing data sets in this paper, the reconstruction image data in step 1 are input into the improved VGG transfer learning model in Section 3.4 for training and testing. Then, a trained improved VGG transfer learning model can be obtained.
- Step 3: For the voltage sag signals to be identified, the corresponding reconstruction images are generated which are input into the trained model in step 2. Finally, the results of voltage sag source recognition are achieved.

In addition, the results of step 3 are added to the historical database for updating.

## 4. Example Analysis

### 4.1. Data Acquisition

**Figure 11:**
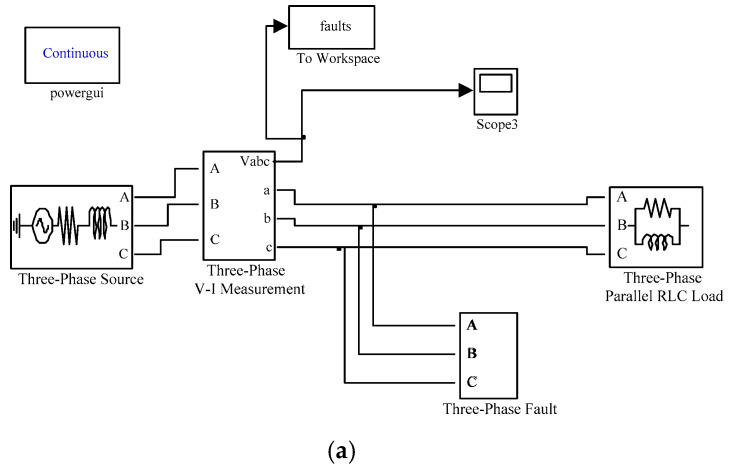

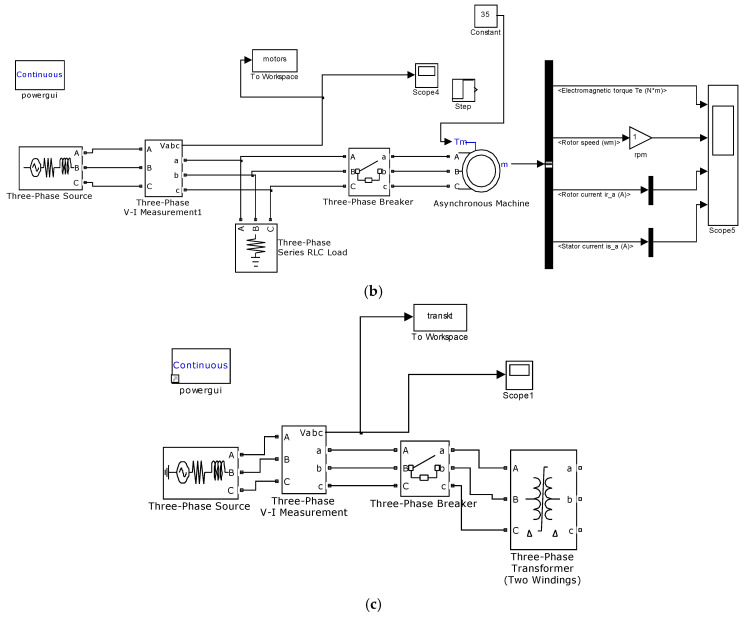
The simulation models of (**a**) short circuit fault, (**b**) large induction motor starting, and (**c**) unloaded transformer energizing in MATLAB/SIMULINK.

The simulation models shown in Figure 11 are built in MATLAB/SIMULINK, and their electrical parameters are changed to obtain various types of voltage sag signal samples at fault points or access points. For short circuit faults, the duration, short-circuit impedance and line loads are changed, and 1000 groups of samples are set for each type, totaling 7000 groups. For the starting of induction motor, the starting time, the internal parameters of the motor and the line load are changed, totaling 1000 groups. For large transformer energizing, the switching time, transformer capacity and connection mode are changed, totally 1000 groups. Because the actual data will be affected by noise, the original 9000 groups of data are superimposed with 20 dB and 10 dB white Gaussian noise, and finally 27000 groups of sample data are obtained. The formula of signal to noise ratio is

(16) $$SNR=10\cdot \mathrm{lg}\frac{P_{s}}{P_{n}}$$

where *P_s_* is the power of sag signal, *P_n_* is the power of noise. The unit of *SNR* is dB. The larger the *SNR* is, the smaller the noise is.

The basic frequency of the simulation model is set to 50 Hz and the total simulation time is set to 1 s. The sampling frequency is set to 1 kHz, so the sampling point of voltage signal is 1000. Voltage sag signal samples are reconstructed by phase space reconstruction to obtain the sample of voltage sag reconstruction image, and the data of the sag reconstruction image is used as the input data set of the model in this paper. 

Four fold cross validation method was used to validate the experiment, that is, 750 groups of samples were selected as training set in each type of sag source, 250 groups of samples were used as test set, and the average of four times of test accuracy was taken as the result.

### 4.2. Analysis of Noise Immunity for Voltage Sag Phase Space Reconstruction

**Figure 12:**
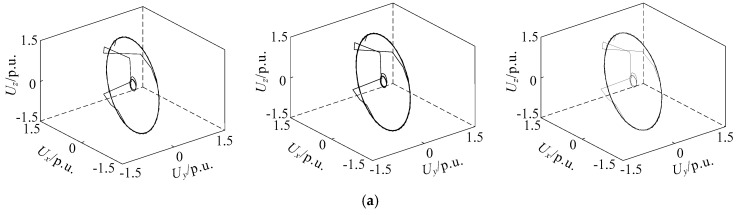

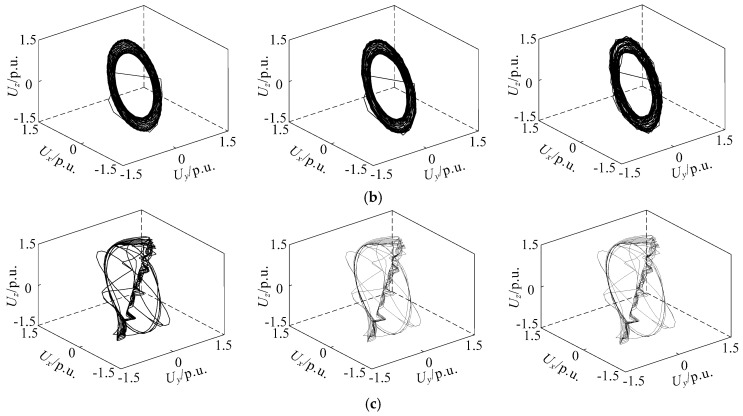
Samples of phase space reconstruction of voltage sag with no noise (**left**), 20 dB (**middle**) and 10 dB (**right**) white Gaussian noise in phase A of (**a**) single phase short circuit fault, (**b**) large induction motor starting and (**c**) unloaded transformer energizing.

Because the actual data will be affected by noise, the original 9000 sets of data are superimposed with 20 dB and 10 dB white Gaussian noise. Three samples of phase space reconstruction image of voltage sag in phase A with 20 dB and 10 dB white Gaussian noise are shown in Figure 12. 

Due to the strong noise, the phase trajectory is not smooth. Except for the type of motor starting, all the phase space reconstruction images are hardly affected. Therefore, the image recognition of voltage sag sources based on phase space reconstruction has a good ability to overcome the noise.

### 4.3. Analysis of Attention Mechanism

**Figure 13:**
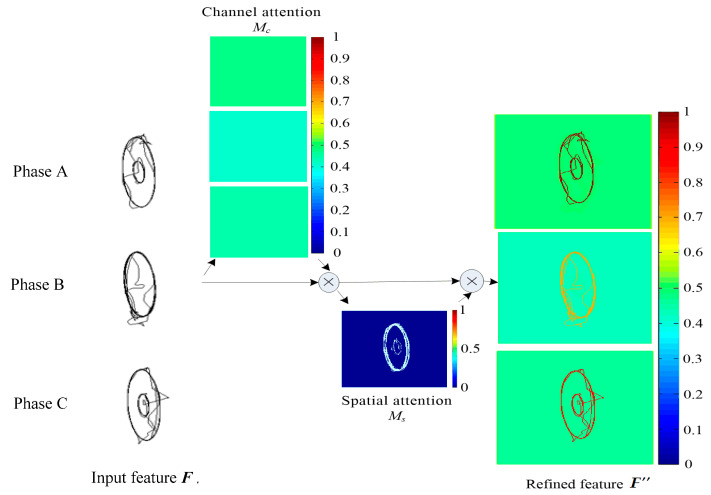
The visualization process of attention mechanism. The channel attention *M_c_*, spatial attention *M_s_* and refined feature ***F*’’** are shown in heat maps.

Attention mechanism can enhance feature extraction, which leads to better classification effect. As can be seen in Figure 13, a sample attention mechanism process of A- and C-phase short circuit type of sag is clearly shown in the form of heat map.

The input feature ***F*** is gray scale reconstruction images of phase A, B, and C. The channel attention, spatial attention and refined feature are shown in visual heat maps, where different colors represent different weights. In the channel attention, phase A is 0.51, phase C is 0.49, and phase B is 0.42, that is, phase A and phase C are more important than phase B. In the spatial attention, the two limit cycles which are 0.37 mean that the sag magnitude is noticed. Combining the channel attention and spatial attention, the refined feature ***F’’*** shows the different weights of three phase’s signal. Phase A is about 0.96 for dark red, phase C is about 0.85 for red, which means that they are the effective information for which type it belong to. Phase B is 0.65 for yellow, which plays the least role in classification. These are consistent with the characteristics of A- and C-phase short circuit fault. Through the attention mechanism, the feature extraction process is strengthened and the final classification results can be more accurate.

Attention mechanism can make the model pay more attention to the effective information, ignore the invalid information, and improve the interpretability of the model. The CBAM attention used in this paper is a combination of channel attention and spatial attention, in which channel attention can indicate which is more important to the results in phase A, B, C, and which pixels and regions in images of phase A, B, C are more important. Combining the two methods can make the model pay more attention to the areas that have great influence on the results, ignore the areas of invalid information, and improve the effectiveness and interpretability of the model.

### 4.4. Analysis of Classification Effect of Feature Vector

Before the output layer, VGG 16 model based on attention mechanism has a fully connected layer with 128 dimensions, which extract 128 final features of sag. These 128 values are characteristic quantities reflecting the characteristics of the reconstructed image itself, which have no practical significance. For example, as shown in Table 1, the feature data within the same class are similar, while the feature data between classes are clearly distinguished.

**Figure 14:**
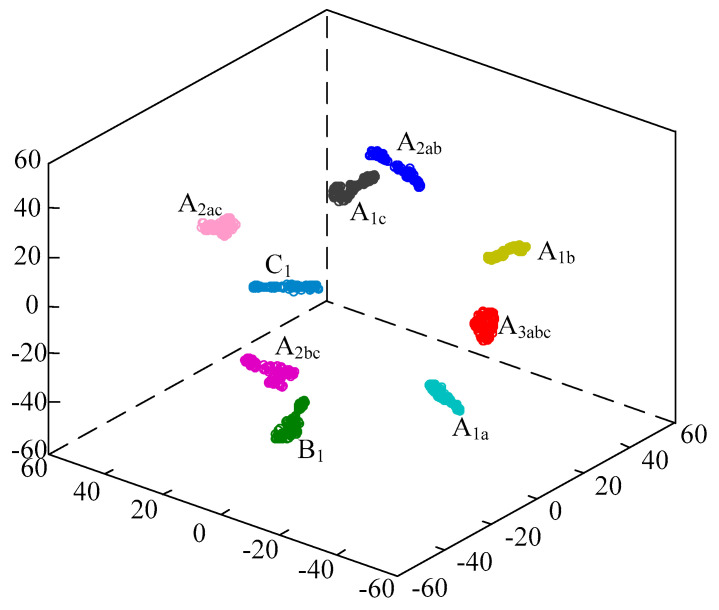
3D projection for feature extraction based on attention-based VGG 16 model.

In order to observe the quality of automatic feature extraction in the proposed model, t-Distributed Stochastic Neighbor Embedding (t-SNE) [35] algorithm can be used to project the extracted feature vectors to three-dimensional space for observation. The three-dimensional projection of the extracted feature vectors can be drawn as shown in Figure 14. It can be seen that the projection boundaries of nine types of voltage sag feature vectors extracted by the proposed model are clear, and the distribution distance between each type is abundant.

### 4.5. Network Training Process and Contrastive Analysis

• Separate training of VGG 16 model

**Figure 15:**
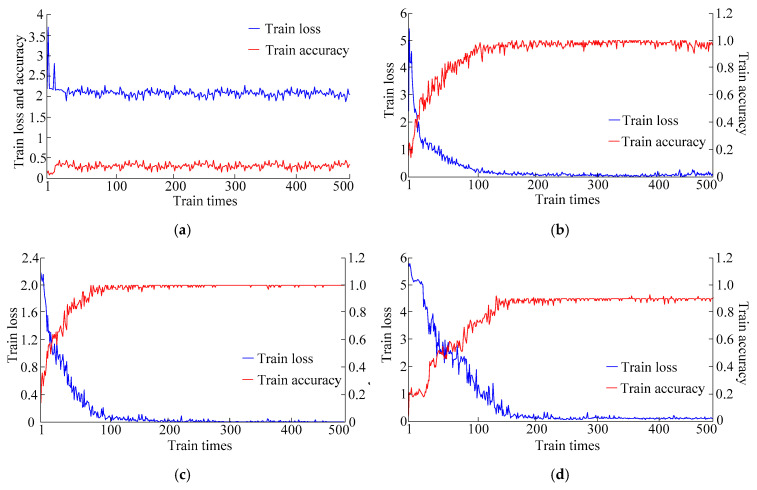
(**a**) VGG 16 model separate training process, (**b**) VGG 16 transfer learning model without attention mechanism combined training process, (**c**) improved VGG transfer learning model combined training process and (**d**) combined training process directly adopting voltage sag signal image.

VGG 16 model is used for separate training, and the pre-trained parameters of ImageNet data set are not used. As shown in Figure 15a, with the increase of iteration times, the loss of the network does not decrease, and the accuracy of the model is less than 50%, the effect is very poor. If VGG 16 model without pre-trained parameters were used to identify voltage sag sources, it would be necessary to increase sample data and training times to achieve good results, which is inefficient and the results are not necessarily ideal. Therefore, it is necessary to introduce the idea of transfer learning to model combined training.

• Combined training of VGG 16 transfer learning model without attention mechanism 

VGG 16 without attention mechanism and standard VGG 16 are used to construct the transfer learning model of VGG 16 without attention mechanism, and combined training is carried out. As shown in Figure 15b, with the increase of the number of iterations, the loss of the network decreases gradually, and the accuracy of the generated model increases gradually which tends to be 96%. However, the stability of network loss and model accuracy is poor. Especially in the late iteration period, the over-fitting phenomenon occurs which leads to the large oscillation.

• Combined training of improved VGG transfer learning model

Attention mechanism is added to construct the improved VGG transfer learning model shown in Section 3.4, and combined training is carried out. In Figure 15c, with the increase of iterations, the loss of the network decreases gradually, and the accuracy of the generated model increases gradually, which tends to be 100%. Moreover, in the late iteration period, the attention mechanism restrains the over-fitting phenomenon, so that the stability of network loss and model accuracy is better.

• Training using voltage sag signal image

Without phase space reconstruction, the improved VGG transfer learning model is trained by using voltage sag signal images as the input directly. As shown in Figure 15d, with the increase of iteration times, the loss of the network decreases and the accuracy of the generated model increases gradually, but the convergence speed is slower than that in Figure 15c, and the accuracy is only about 90% which is lower than the result of reconstruction sample by phase space reconstruction technology. This is because the instantaneous waveforms of voltage sag voltage signals are dense, and the difference between the signal images of different sag sources is not obvious in the long-term signal images.

### 4.6. Result Analysis

In order to evaluate the recognition results of each type of sag source, *Accuracy* and *F*1 [36] are selected as the evaluation indexes. Among them, *Accuracy* is a single index and *F*1 is a comprehensive index.

(17) $$Accuracy=\frac{C}{T}$$

(18) $$Precision=\frac{C}{P}$$

(19) $$F1=2\times \frac{Precision\times Accuracy}{Precision+Accuracy}$$

where *C* is the number of samples correctly recognized for a certain type of voltage sag sources, *T* is the number of samples that are true for this type, *P* is the number of all samples recognized as this type, *Accuracy* is the ratio of the number of samples correctly recognized for this type to the number of samples that are true for this type, *Precision* refers to the ratio of the number of samples correctly recognized for this type to the number of all samples recognized as this type.

The identification results of voltage sag sources are shown in Table 2. From Table 2, it can be seen that the average recognition *Accuracy* and *F*1 of the reconstruction image of the noise-free voltage sag signal are 100%, which indicates the feasibility of image recognition of voltage sag sources. With the increase of noise, the average recognition *Accuracy* decreases to 98.3% and the *F*1 decreases to 95.4%, which still remain at a high level and reflect the anti-noise ability of this method. 

From the point of view of practical application, the average recognition *Accuracy* and *F*1 of this method are high for the single phase short circuit fault sag source type which accounts for a large proportion in power system. Among them, the average recognition *Accuracy* and *F*1 of A_1a_ type is 99.4% and 98.2% respectively. The average recognition *Accuracy* and *F*1 of A_1b_ type is 99.2% and 98.2% respectively. The average recognition *Accuracy* and *F*1 of A_1c_ type is 99.5% and 97.9% respectively.

The methods of reference [4,5,15], and [17] (hereinafter referred to as method 1, method 2, method 3, and method 4) are compared with the experimental results of voltage sag source identification method based on phase space reconstruction and improved VGG migration learning proposed in this paper, as shown in Table 3 and Table 4.

Table 3 shows that the recognition accuracy of the proposed method for different voltage sag sources is relatively high. This is because the attention mechanism is added in the process of automatic feature extraction, which improves the expression ability and generalization ability of the model. From Table 4, it can be seen that the convergence rate of this method is faster, because the transfer learning method reduces the training cost and improves the training efficiency.

## 5. Conclusions

In this paper, a method of voltage sag source identification based on phase space reconstruction and improved VGG transfer learning is proposed. The effectiveness and superiority of the proposed method are verified by an example, which is mainly embodied in the following aspects:

- Voltage sag signal image is reconstructed into phase space image, which not only retains the complete characteristics of sag, but also has more intuitive and concise image features.
- Attention mechanism is added to VGG model to further automatically extract image features to prevent over-fitting. It has an excellent classification effect and improves the accuracy of model recognition.
- The idea of transfer learning is introduced to train the network on the basis of other image classification results, which improves the efficiency of network training.

The basis of image recognition is image accuracy. In this paper, only voltage sag signals of one single sampling frequency are identified as voltage sag sources. How to ensure the accuracy of voltage sag reconstruction under different sampling frequencies is the next research direction.

## Figures and Tables

**Table 1:** 128-dimensional feature vectors of the features for different types of voltage sags.

Dimension	1	2	3	4	5	6	7	8	…
**A _1a_**	4.574108	0.630084	0	0	5.079928	0	4.930518	0	…
	4.555246	0.635833	0	0	5.045293	0	4.893074	0	…
	4.459694	0.652800	0	0	4.856162	0	4.799524	0	…
**A_1b_**	3.613004	5.856254	0	0	0.665880	0	0	0	…
	3.523590	5.643432	0	0	0.597539	0	0	0	…
	3.550356	5.732181	0	0	0.459643	0	0	0	…
**A_1c_**	2.309263	0.511907	0	0	5.853089	3.568975	2.278446	0	…
	2.218758	0.484885	0	0	5.708840	3.486815	2.193294	0	…
	2.673487	0.980059	0	0	6.963194	4.116153	2.532054	0	…
**A_2ab_**	4.865230	1.006909	0	0	0	0	2.188855	0	…
	4.929275	1.037814	0	0	0	0	2.162757	0	…
	4.954888	1.041241	0	0	0	0	2.105883	0	…
**A_2ac_**	3.729304	1.305431	0	0	3.439787	0.934509	1.063446	0	…
	3.763325	1.190667	0	0	3.463085	1.109307	1.093839	0	…
	3.919007	1.267426	0	0	3.706130	1.129699	1.240683	0	…
**A_2bc_**	1.402077	0.637477	0	0	0	0	0	0	…
	1.228110	0.477076	0	0	0	0	0	0	…
	1.315113	0.497585	0	0	0	0	0	0	…
**A_3abc_**	3.255797	0	0	0	0.732882	2.763226	0	0	…
	3.085777	0	0	0	0.683569	2.616498	0	0	…
	3.267628	0	0	0	0.797595	2.761694	0	0	…
**B_1_**	0	3.584848	0	0.130128	1.752869	0	0	0	…
	0	3.790805	0	0.230235	1.670009	0	0	0	…
	0	3.980163	0	0.123113	1.961738	0	0	0	…
**C_1_**	0	2.755503	0	0	0	0	0	0	…
	0	2.504199	0	0	0	0	0	0	…
	0	2.720874	0	0	0	0	0	0	…

**Table 2:** The identification results of voltage sag sources.

Sag Source Type	*Accuracy*/%			*F*1/%		
	Noise-Free	20 dB	10 dB	Noise-Free	20 dB	10 dB
**A_1a_**	100	100	98.4	100	98.7	95.8
**A_1b_**	100	99.6	98.0	100	98.8	95.8
**A_1c_**	100	100	98.4	100	98.6	95.1
**A_2ab_**	100	99.8	99.6	100	96.8	93.9
**A_2ac_**	100	100	98.4	100	95.8	93.7
**A_2bc_**	100	99.2	98.4	100	96.9	95.9
**A_3abc_**	100	100	97.2	100	99.3	93.8
**B_1_**	100	100	98.8	100	99.6	96.8
**C_1_**	100	100	99.2	100	99.9	96.8

**Table 3:** Comparison of the recognition accuracy of voltage sag sources.

Sag Source Type	*Accuracy*/%				
	Method 1	Method 2	Method 3	Method 4	Proposed Method
**A_1a_**	99.4	97.8	96.5	93.4	99.5
**A_1b_**	99.6	98.2	96.3	93.6	99.2
**A_1c_**	98.5	97.1	95.2	94.1	99.5
**A_2ab_**	98.9	97.8	96.4	94.2	99.8
**A_2ac_**	98.1	97.6	96.9	93.6	99.6
**A_2bc_**	99.3	97.9	97.1	93.8	99.2
**A_3abc_**	99.2	98.2	96.3	94.5	99.1
**B_1_**	99.2	98.3	97.2	94.2	99.6
**C_1_**	99.6	98.6	96.8	93.6	99.7

**Table 4:** Comparison of convergence rate of voltage sag source recognition model.

	Method 1	Method 2	Method 3	Method 4	Proposed Method
**Train times**	416	465	614	750	305
